# Deceptive illusory cues can influence orthogonally directed manual length estimations

**DOI:** 10.3758/s13414-024-02991-7

**Published:** 2025-01-13

**Authors:** Shijun Yan, Jan M. Hondzinski

**Affiliations:** 1 School of Kinesiology, Louisiana State University, 1250 Huey P. Long Field House, 50 Field House Drive, Baton Rouge, LA 70803 USA; 2 Shirley Ryan AbilityLab, Northwestern University, 355 E Erie St, Chicago, IL 60611 USA; 3 Department of Physical Medicine and Rehabilitation, Northwestern University, 355 E Erie St, Chicago, IL 60611 USA

**Keywords:** Motor control, Perception and action, Visual perception

## Abstract

We examined participants’ abilities to manually estimate one of two perpendicular line segment lengths using curved point-to-point movements. Configurations involved symmetrical, unsymmetrical, and no bisection in upright and rotated orientation alterations to vertical-horizontal (V-H) illusions, where people often perceive longer vertical than horizontal segments for equal segment lengths. Participants used two orthogonally directed movements for length estimations: positively proportional (POS) – where greater fingertip displacement involved longer length estimation between configuration intersection start position and fingertip end, and negatively proportional (NEG) – where greater fingertip displacement from the screen edge start position toward configuration intersection involved a shorter length estimation between configuration intersection and fingertip end. Length estimations followed most standard perceptual aspects of the V-H illusion for POS estimations, yet differed between upright and rotated orientations for the symmetrical configuration. NEG estimations revealed no illusory influences. Use of allocentric programming likely accompanied POS estimations to explain V-H illusory influences on perceptuomotor control.

## Introduction

People show the highest endpoint accuracy when they receive visual feedback of the hand and target during reaching movements (Beaubaton & Hay, 1986) to suggest an important role of vision for such control. Allocentric environmental cues can also alter the accuracy of goal-directed movements (Blouin et al., 1993; Gentilucci et al., 1997; Hondzinski & Cui, 2006). Using a grid-pattern background can benefit endpoint accuracy of pointing (Coello & Grealy, 1997; Conti & Beaubaton, 1980; Schoumans et al., 2000), while using deceptive patterns containing biased information can degrade endpoint accuracy and increase errors (e.g., Heath & Binsted, 2007). Visual illusions, which bias size estimations of the object or portions of the object, including magnitude of a line length or circle diameter, can deceive people to degrade endpoint accuracy in an unwanted manner for targeted movements (Charras & Lupiáñez, 2010; Wolfe et al., 2005; Yan & Hondzinski, 2021; Yan et al., 2023) or in a desirable manner for greater toe clearance when ascending stairs (Foster et al., 2015; Skervin et al., 2021), a step (Elliott et al., 2009), or stepping over a low-height object (Foster et al., 2016; Sakurai et al., 2024). Use of visual illusions also provides a potential avenue to employ preferred implicit over explicit mechanisms for motor learning (Facchin et al., 2021). However, discrepancies among previous studies make it difficult to determine how people will react when presented with visual illusions.

Some people produce movements corresponding to visual perceptions, while others do not. Accurate movements can subsist with inaccurate estimates based on illusory perceptions to reveal a dissociation of vision for perception and action to support the “two-visual-system” hypothesis, defined as two distinct visual neural pathways: one for perception and one for action (Goodale & Milner, 1992). Studies in which people adjusted their hand aperture to match the diameter of a circular object without size induced bias from a visual illusion (Aglioti et al., 1995; Ganel et al., 2008; Haffenden & Goodale, 1998) provide support for this hypothesis. In contrast, inaccurate movements according to illusory perceptions can also exist to reveal an association between vision for perception and vision for action. Studies in which people naturally altered their vertical toe clearance according to real-time illusory perceptions of line segment lengths during stepping (Elliott et al., 2009; Foster et al., 2015, 2016; Sakurai et al., 2024; Skervin et al., 2021) provide support against the two-visual-system hypothesis and the dissociation between perception and action (Franz & Gegenfurtner, 2008; Franz et al., 2000; Kopiske et al., 2016, 2017; Mendoza et al., 2005). People may use scene-based or allocentric frames of reference which bias perceptual judgments according to the visual illusion and effector-based (i.e., a body segment such as a finger) or egocentric frames of reference which eliminate visual illusory perceptual influences on action (Goodale, 2014; Schenk, 2006), potentially explaining the differences between viewpoints. The separate use of allocentric or egocentric frames of reference, which might seem obvious for stepping without directly watching the feet or pointing using the unseen hand (Schenk, 2006), remains ambiguous for limb control of the seen hand, in which evidence supports their simultaneous use (Carrozzo et al., 2002; Gentilucci et al., 1997; Hondzinski & Cui, 2006). The simultaneous use of allocentric and egocentric frames of reference would also explain illusory influences over manual length estimations during a finger-fixation condition, where participants directed their gaze toward their finger during task performance (Yan & Hondzinski, 2021). Thus, one aim of this study was to try to understand the potential subtle task differences that may explain discrepancies in the literature for visual perception use for action of upper limb movements with emphasis on the seen hand.

Defining a task as perceptual or action based is fundamental when assessing vision for action. Some researchers consider manual estimations, using grasp aperture, a perceptual task, and/or measure because participants adjust the distance between the index finger and thumb to directly match the perceived object size, diameter, or length. Explanations from these studies for perceptual, thus potential illusory influences, on actions include vision stored for off-line movement control, use of pantomime gestures without haptic feedback of an object, and engagement of specific brain areas (summarized in Goodale, 2014). However, other researchers question the interpretation of manual estimation using grasp aperture as purely a perceptual measure, as it requires a motor/action component, thus more than a judgment of visual dimension (i.e., Franz, 2003; Ganel et al., 2012; Kopiske et al., 2016). Manual length estimations other than the use of grasp aperture also exist and can reveal discrepancies for the use of visual illusory influences for action of upper limb movements.

**Fig. 1:**
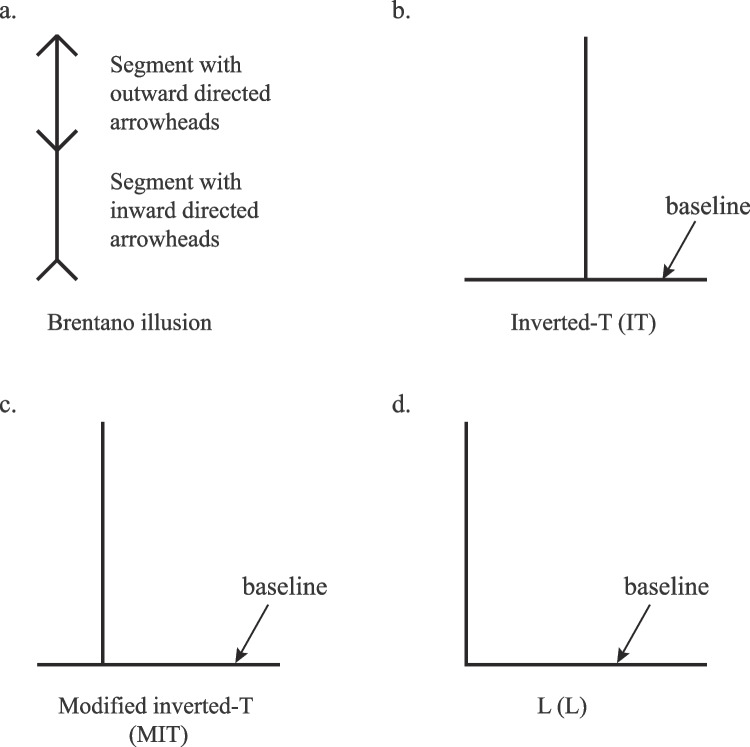
Brentano illusion (**a**) and upright vertical-horizontal (V-H) illusions (**b**,** c**, and **d**). Different configurations of the V-H illusion were presented to participants for perceptual task in the present study: IT (**b**), MIT (**c**), and L (**d**). Horizontal (baseline for upright configurations) and vertical segments for each stimulus are equal in length, yet the vertical segment appears longer to many people, especially for IT. Same configurations with 90° clockwise rotation were also used in the perceptual task. *MIT* modified inverted-T, *IT* inverted-T

Drawing point-to-point movements on the same surface can result in endpoint errors according to a perceived shorter or longer segment length of the Brentano illusion (Fig. 1a; de Grave et al., 2004). While viewing line segments with inward- or outward-directed arrowheads, people asked to move a pen tip along the flat surface and in a direction parallel to the given segment overshot the segment end with outward-directed arrowheads and undershot the segment end with inward-directed arrowheads. Thus, participants’ manual length estimations mirrored visual perceptions according to the illusion when movement direction paralleled the line segment of the illusion. Conversely, illusory effects on segment length estimations disappeared when the participants moved the pen tip along the flat surface in a direction perpendicular to the given line segment of the illusion. Clearly, illusory influences on manual estimations of segment lengths using point-to-point straight, one-dimensional (1D) movements can depend on movement direction. Knowing that amplitude control can also depend on movement direction (Bock & Arnold, 1993; Hening et al., 1988; Hondzinski & Cui, 2006; Soechting & Flanders, 1989a, b), we considered a visual illusion with influences on manual estimations of segment lengths in more than one direction/dimension.

In one version of the vertical-horizontal (V-H) illusion, the vertical bisecting segment of an inverted-T (IT) usually appears relatively longer than the horizontal bisected segment of equal length (Fig. 1b; Mamassian & de Montalembert, 2010; Masin & Vidotto, 1983; Vishton et al., 1999; Wolfe et al., 2005). When asked to estimate a given length of a horizontal or vertical segment to complete an IT configuration, people overestimated the length of the vertical segment and underestimated the length of the horizontal segment when using a computer mouse to draw the missing orthogonal component (Gavilán et al., 2017). Manual length estimations of the bisecting segment of upright and 90° counterclockwise-rotated IT configurations also showed overestimations for single motion, oppositely directed, naturally curved thus two-dimensional (2D), point-to-point movements when gaze was only directed toward the movement of the finger or only toward the configuration (Yan & Hondzinski, 2021). When these participants concentrated on the configuration prior to movement and the movement during task performance under a free-gaze condition, it led to attenuation of the overestimations and better accuracy. These results revealed that deceptive V-H visual illusory cues can generally guide upper limb movements given certain performance parameters even in cases in which the actual curved-path 2D movement does not directly match the length estimation. Curved-path point-to-point movements, in this case, indicate lifting the finger to move from the start point to the end point, instead of using a sliding motion while maintaining the fingertip on a flat surface. In each of these studies, mouse drawing (Gavilán et al., 2017) or curved-path point-to-point movements (Yan & Hondzinski, 2021) occurred in a blank space and length estimations were positively proportional to segments lengths so that longer movement amplitudes corresponded to longer length estimations. In the present study, we chose to determine whether illusory influences would exist for positively and negatively proportional manual length estimations of the bisected segment of the V-H illusion. Negatively proportional estimations entail inverse relationships between movement amplitude and length estimations. Rather than using a blank space, we accounted for potential directional influences by requiring estimations on an extended segment of V-H configurations.

We considered that the two primary explanations used to explain V-H illusory perceptual judgments might also explain perceptuomotor control outcomes in this study. One explanation is that perceived segment lengths depend on alterations of the elliptical visual field (Künnapas, 1957b). The location of two eyes in humans provides a horizontally oriented elliptical visual field. When viewing an IT configuration, the shorter distances from the ends of the vertical segment to the boundaries of the upper and lower visual field and the longer distances from the ends of the horizontal segment to the right and left visual field boundaries make the vertical segment appear longer than its actual length. The perceived longer vertical segments can also occur for IT with 90° rotations slightly attenuating illusory overestimation of the vertical segment, supporting the visual field explanation (Charras & Lupiáñez, 2010; Renier et al., 2006). Slight attenuation of the illusion magnitude for 90°-rotated IT supports the second explanation of V-H illusion on perceptual judgments (Charras & Lupiáñez, 2010; Finger & Spelt, 1947; Künnapas, 1955a; Mamassian & de Montalembert, 2010; Wolfe et al., 2005). The second explanation involves bisection influences, in which the bisected segment appears shorter than the bisecting segment in the IT configuration (Künnapas, 1955a). The illusory extent can be reduced by changing the location of the bisecting segment to an unsymmetrical position (see, for instance, Fig. 1c for a modified inverted-T (MIT)) or with no bisection (see Fig. 1d for L), so that the symmetrical bisecting IT produces the strongest illusion to support bisection influences on perceptual judgments (Mamassian & de Montalembert, 2010; Wolfe et al., 2005). Since illusory influences across different configurations of the V-H illusion for upright and rotated orientations for manual estimations are unknown, we included them in the present study.

In this study we wanted to determine whether illusory influences would exist for positively and negatively proportional manual estimations of one segment length of upright and rotated V-H illusion-like configurations. Participants always estimated the length of a baseline segment orthogonal to their movement, and movement direction varied according to orientation of the configuration. We expected that participants would use allocentric and egocentric frames of reference (Carrozzo et al., 2002; Gentilucci et al., 1997; Hondzinski & Cui, 2006; Yan & Hondzinski, 2021), which depend on task. We hypothesized that tasks encouraging greater use of allocentric frames of reference, such as positively proportional estimations, would produce a greater illusory bias on manual estimations than negatively proportional estimations to contest the two-visual-system hypothesis. We also hypothesized that manual estimations for different V-H illusion configuration symmetries and configuration orientations would be greatest for the upright IT, in which visual field and bisection explanations complement each other.

## Methods

### Participants

Participants read and signed informed consent prior to participation, which was approved by the University’s Institutional Review Board. Pilot data revealed moderate to high effect sizes and greater than 80% power on main effects for 12 participants. Thus 12 right-handed young adults (four females; age = 23 ± 4.9 years; height = 174.7 ± 8.6 cm) recruited from Louisiana State University participated in this study. Right-hand dominance was determined using the Edinburgh Handedness Inventory; range 70–100 (Oldfield, 1971). Participants had no difficulty viewing visual stimuli on the computer screen within arm’s length (visual acuity 20/25 or better on the Snellen eye chart assessment).

### Setup

**Fig. 2:**
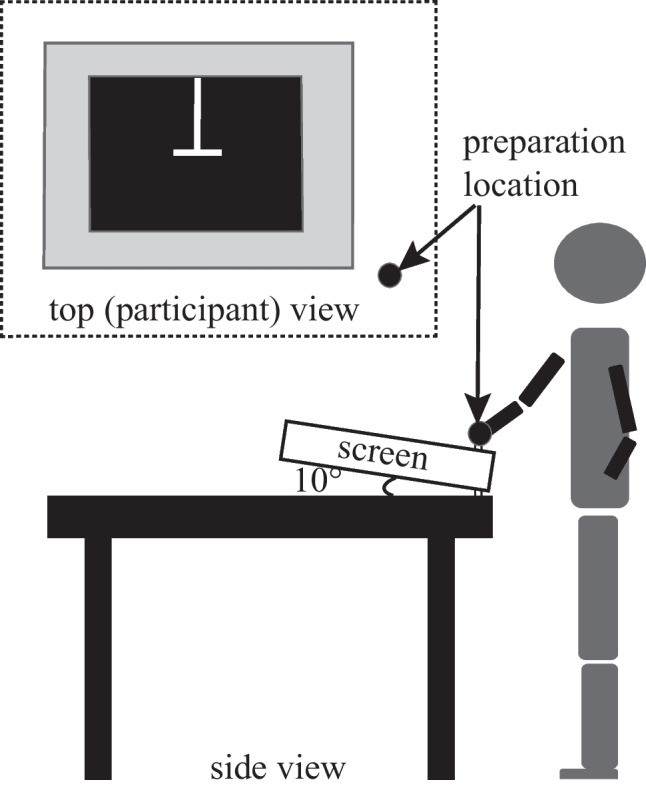
Side view of experiment setup. Insert involves an overhead view of the screen and start position from the participants’ perspective

Figure 2 shows the experimental setup and preparation location. The slightly tilted computer screen (10° from horizontal) helped avoid reflective interference with data collection and made for easier viewing. Participants stood in front of the front edge of a 10.5-cm black square frame surrounding a 27.5-cm square computer screen to help avoid illusory influences that can accompany changes in the surrounding frame, even though it might reduce the perceptual effect (Gavilán et al., 2017; Houck et al., 1972; Künnapas, 1959). Feet locations at a self-selected distance apart were marked to remain constant across trials. Distance between each participant and the near edge of the frame was 10 cm. Eye height across participants was 66 ± 8.2 cm above the front edge of the computer screen. Shorter participants chose to stand on an elevated surface for better view of the screen and greater reaching comfort during movements. The computer screen resolution of 1,280 × 1,024 was set at medium for brightness. Participants reported no difficulty viewing white targets on the black background or reaching to various locations on the screen.

### Perceptual judgment stimuli

We included a relative size judgment task to determine if participants were influenced by the V-H illusion perceptually. As a reminder, people often report that the length of the vertical segment exceeds the corresponding length of the horizontal segment when segments of the V-H illusion are actually equal in length. Configurations for the perceptual task included symmetrical bisection with an inverted-T (IT), unsymmetrical bisection with a modified inverted-T (MIT), and no bisection with an L (see Figs. 1b, c, and d, respectively). Stimuli were 3-point width (1.6 mm) solid white line segments presented on a black background designed using Microsoft PowerPoint (Office 2010). Configurations were presented upright or rotated 90° clockwise. The V-H intersection of all configurations was placed at the center of the square screen. We altered configuration baseline segment lengths (45 mm, 60 mm) for variety. The visual angle for 45 mm and 60 mm was approximately 4° and 5.3°, respectively. The horizontal segment was the baseline segment for upright configurations and the vertical segment was the baseline segment for rotated configurations. The length of the non-baseline segment included equal to, 10% increase in, and 10% decrease in the baseline reference segment. The choice of ± 10% was less than the 16% (Blanuša & Zdravković, 2015; Mamassian & de Montalembert, 2010) and 20% (Charras & Lupiáñez, 2010; Vishton et al., 1999) estimated length differences used to perceive equal vertical and horizontal segment lengths of the V-H illusion, produced greater illusory responses than 15% during pilot testing, and allowed us to make direct comparisons to others in the literature (e.g., Chouinard et al., 2017).

### Perceptual judgment protocol

Participants were presented with one configuration at a time in a randomly determined order. After viewing a configuration, participants orally reported “equal” when the segments appeared identical in length; “horizontal” when the horizontal segment appeared longer than the vertical segment; and “vertical” when the vertical segment appeared longer than the horizontal segment. Participants were presented with three trials of each configuration (IT, MIT, L), baseline length (45 mm, 60 mm), orientation (upright, rotated), and relative length (equal, longer horizontal segment, longer vertical segment) to obtain 36 trials for IT, MIT, and L and a total of 108 trials. Three breaks were given to each participant for as long as requested. Duration of the task did not exceed 25 min.

### Perceptuomotor stimuli

**Fig. 3:**
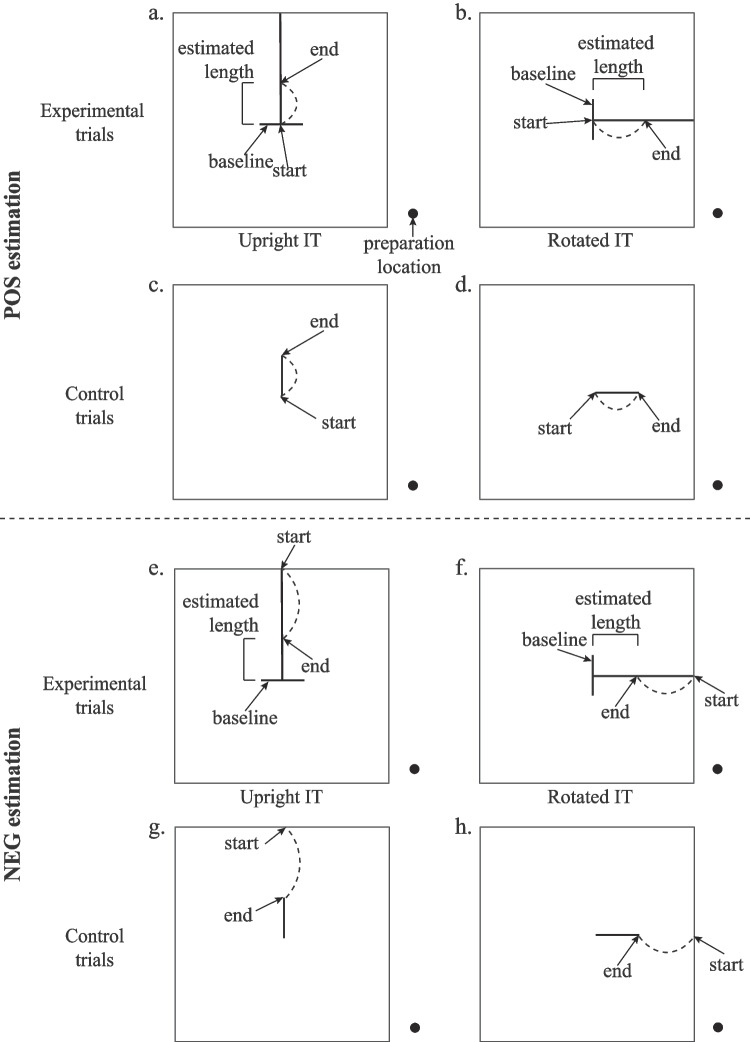
Examples of inverted-T (IT) stimuli for the perceptuomotor task are presented from an overhead view. Participants estimated horizontal baselines using positively proportional (POS) (**a**) and negatively proportional (NEG) (**e**) estimations for upright configurations and estimated vertical baselines using POS (**b**) and NEG (**f**) estimations for rotated configurations. Control trials for each condition are shown (**c**, **d**, **g**, and **h**). Preparation location (shown as a black dot) was towards the right, lower edge of the screen. Dashed curves represent the curved-path start touch-lift-end touch movements performed. Presentations for MIT and L configurations were similar but are not depicted here

Stimuli included versions of IT, MIT, and L with either 45-mm or 60-mm baseline lengths and were projected to the computer screen using PowerPoint presentation mode. Use of two lengths prevented memorization of one movement distance. V-H intersections of each configuration were set at the center of the square screen and stimuli involved 3-point width (screen projected line width = 1.6 mm) solid white line segments on a black background. Horizontally (Fig. 3a, c) and vertically (Fig. 3b, d) oriented reference segments (baselines) intersected with a perpendicular segment (non-baseline) extended to the edge of the computer screen. The visual angle for the extended segment in configurations of experimental trials was approximately 12° for both sizes. Use of the non-baseline segment for all configurations allowed us to provide directional assistance on the computer screen. Therefore, configurations were oriented upright or rotated 90° to the right (clockwise) so that the horizontal or vertical segment could be used as the baseline reference and movements for length estimations could occur primarily in the vertical (anterior–posterior) or horizontal (medial–lateral) directions.

### Perceptuomotor protocol

Participants began with their right fingertip of the index finger on the preparation location (black dots, Figs. 2 and 3), 7.5 cm to the right of and 6 cm anterior to the front edge of the screen setup (frame not shown in Fig. 3). An investigator manually advanced from a blank slide to a slide containing one of the stimuli subsequent to ensuring the participant was ready. After viewing a configuration, participants moved their fingertip from the preparation location to the start position, then manually estimated the length of the baseline segment using a touch-pause-lift-touch curved finger movement. They received instructions to initiate comfortably paced movements soon after a stimulus appeared on the screen. After a few practice trials, participants reacted within about 1 s after stimulus presentation and seemed to move in a comfortable manner as requested. Protocol included two estimation types of baseline lengths. Participants used positively and negatively proportional movements.

For positively proportional movements (POS), participants moved their fingertip from the preparation location to the intersection of the vertical and horizontal segments of the configuration upon stimulus presentation (first touch/start), paused for 1 s, then lifted the finger to move it along a curved path to an end location along the extended segment (second touch/end), so that the distance from start to end locations matched the estimated length of the baseline. After pausing about 1 s at the end location, the investigator removed the stimulus from view by advancing to a blank slide and provided a “relax” command, which signaled the participant to move their finger back to the preparation location and prepare for the next trial. In POS estimation, participants made movements away from the body, primarily in an anterior direction for configurations with a horizontal baseline segment (Fig. 3a) and rightward/lateral movements for configurations with a vertical baseline segment (Fig. 3b), so that making a longer movement corresponded to a longer length estimation. In control trials, participants touched the endpoints of a single vertically (Fig. 3c) or horizontally (Fig. 3d) oriented segment of 45 mm or 60 mm using the same touch-pause-lift-touch technique. Note that the first touch always corresponded to the end of the vertical segment closer to participants for upright configurations or to the left end of the horizontal segment closer to a participant’s midline for rotated configurations.

For negatively proportional movements (NEG), participants moved their fingertip from the preparation location to the intersection of the extended segment with the screen edge upon stimulus presentation (first touch/start), paused for 1 s, then lifted the finger to move it to an end location along the extended segment (second touch/end), so that the distance from the V-H intersection to the end location matched the estimated length of the baseline. In this NEG estimation, participants made movements toward their bodies, primarily in a posterior direction, for configurations with a horizontal baseline segment (Fig. 3e) and leftward/medial movements for configuration with a vertical baseline segment (Fig. 3f), so that making a longer movement corresponded to a shorter length estimation. Again, advancing to a blank slide and a “relax” command signaled participants to move back to the preparation location and prepare for the next trial. In a control trial, a single horizontal or vertical segment of 45 mm or 60 mm was presented. Participants touched the intersection of the extended segment with the screen edge (see start position in Figs. 3e and f), then lifted the finger and touched the top or right end of the vertical (Fig. 3g) or horizontal (Fig. 3h) control segments, correspondingly, using the same touch-pause-lift-touch technique previously described.

The perceptuomotor task involved 12 trials in each condition and included: 45 mm IT horizontal baseline (1); 45 mm IT vertical baseline (2); 45 mm MIT horizontal baseline (3); 45 mm MIT vertical baseline (4); 45 mm L horizontal baseline (5); 45 mm L vertical baseline (6); and another six similar conditions for the 60-mm baseline. Four control trials were performed for each configuration symmetry and orientation, baseline length, and estimation type. Half the participants completed POS estimations first and the NEG estimations second, while the other half completed the NEG estimations first and the POS estimations second, while presentation of configuration symmetry and orientation and baseline length were randomized. Participants performed several practice trials to become familiar with each task. Each participant performed a total of 144 trials, which were pseudorandomized prior to the experiment to ensure that no more than two of the same configuration in a given orientation were presented in a row. Breaks were given every 2 min for as long as requested, which was less than 2 min. Duration of the task did not exceed 90 min.

### Data collection and analysis

#### Perceptual judgment task

For the perceptual judgment task, two investigators recorded participants’ responses to maintain accurate reporting and avoid missed reports that occurred a few times during pilot testing. We counted the number of trials that the participants judged correctly and incorrectly, then used the incorrect responses to calculate a percentage based on illusory or opposite illusory effect for each condition and configuration. A correct response referred to a participant reporting a longer vertical segment when the vertical segment was longer, for example, while an example of an incorrect response referred to a participant reporting a longer vertical segment when the horizontal segment was longer. When configurations are presented upright, the bisection and visual field explanations for the V-H illusion coincide with each other and a correct response when vertical segment lengths exceed horizontal segment lengths. Thus, illusory effects only referred to conditions in which participants reported “vertical” or “equal” when viewing a configuration where the horizontal segment length was longer than the vertical segment length or reported “vertical” when viewing a configuration where the horizontal segment length equaled the vertical segment length. Opposite illusory effects referred to conditions where participants reported “horizontal” or “equal” when viewing a configuration where the vertical segment length was longer than the horizontal segment length or reported “horizontal” when viewing a configuration in which the horizontal segment length equaled the vertical segment length. For rotated configurations, illusory effects differed. In the rotated condition, responding “horizontal” or “equal” when viewing an IT or MIT configuration with a longer vertical segment and responding “horizontal” for an IT or MIT configuration with equal vertical and horizontal segment lengths provides support for bisection effects. Support for bisection influences would also correspond to the greatest illusory influences for IT, then MIT, followed by L. Reporting “vertical” or “equal” for all configurations with a longer horizontal segment length or “vertical” for equal vertical and horizontal segment lengths provides support for visual field illusory influences. Responding “horizontal” for an IT or MIT configuration with a longer horizontal segment length corresponds to a correct response that also supports bisection effects.

Percentages of perceptual judgment responses were determined for each participant according to correct and incorrect responses for vertical segment length greater than, equal to, or less than the horizontal segment. Incorrect responses were separated further into illusory and opposite illusory percentages for upright configurations, while incorrect responses for rotated configurations were split into visual field and bisection percentages. Mean percentages were plotted for visual comparisons. Quantitative comparisons among correct perceptual judgment percentages were determined for equal vertical and horizontal segment lengths. Use of equal length responses allowed for complete differentiation among correct and illusory responses while accounting for configuration symmetry and orientation. Normal distributions were assessed using the Shapiro–Wilk W test. We used Friedman ANOVAs (Wilcoxon matched pairs post hoc tests with Bonferroni correction) to identify significant differences across configurations within a given orientation and between orientations for each configuration (α = 0.05 prior to corrections).

#### Perceptuomotor task

For the perceptuomotor task, a 2-cm diameter reflective marker was placed on the fingertip of the right dominant hand and recorded at 60 Hz with a four-camera Qualisys system (Qualisys Medical AB, SE). The 2-cm diameter marker covered the fingertip, was visible on cameras, and participants recruited in pilot testing and the study felt that they could accurately place their fingertip where they wanted. Position data of the fingertip marker were low-pass filtered using a Butterworth second-order filter with 6-Hz cutoff frequency, and differentiated with respect to time to obtain velocity profiles. Start and end of movements were determined when velocity was maintained below 5% of peak velocity for ≥ 100 ms before and after movement, respectively, similar to elsewhere (Adamovich et al., 2001; Yan & Hondzinski, 2021).

Trial-length estimations were needed to determine the primary variable of interest, a displacement ratio (trial length estimation/mean control displacement), which allows for determination of a relative change (for several examples, see summary in Gegenfurtner & Franz, 2007) of interest to account for potentially different pointing strategies used by participants in this study. Displacement ratio represented normalized data to account for two baseline lengths and for the small difference between fingertip and marker location (e.g., Yan & Hondzinski, 2021). For POS estimations, trial length estimation equaled the finger displacement between start and end positions (Figs. 3a and c). Trial length estimation of the NEG estimation trials was determined as the difference between extended segment length (137.5 mm, about 12º visual angle) and the finger displacement between start and end positions (Figs. 3e and g). This allowed for direct comparisons between estimation types. Displacement ratios exceeding 1 represent manual length estimates longer than control trials, thus an underestimation of line segment lengths, while displacement ratios less than 1 represent manual length estimates shorter than control trials, thus overestimation of line segment lengths. Mean displacement ratios were determined for configuration symmetry (IT, MIT, L), configuration orientation (upright, rotated), and estimation type (POS, NEG). Normal distributions were assessed using the Shapiro–Wilk W test. Effects of configuration symmetry, configuration orientation, and estimation type on mean displacement ratio were analyzed with repeated-measures ANOVAs (Tukey’s post hoc tests, α = 0.05) to make direct comparisons between estimation type while accounting for configuration symmetry and orientation. Effect size (ES) corresponding to partial eta squared provided insight into the strength of relationships for significant outcomes. Strength of ES was considered small ≤ 0.25 and large ≥ 0.40, with medium between 0.25 and 0.40 (Cohen, 1969).

## Results

### Perceptual judgment task

**Fig. 4:**
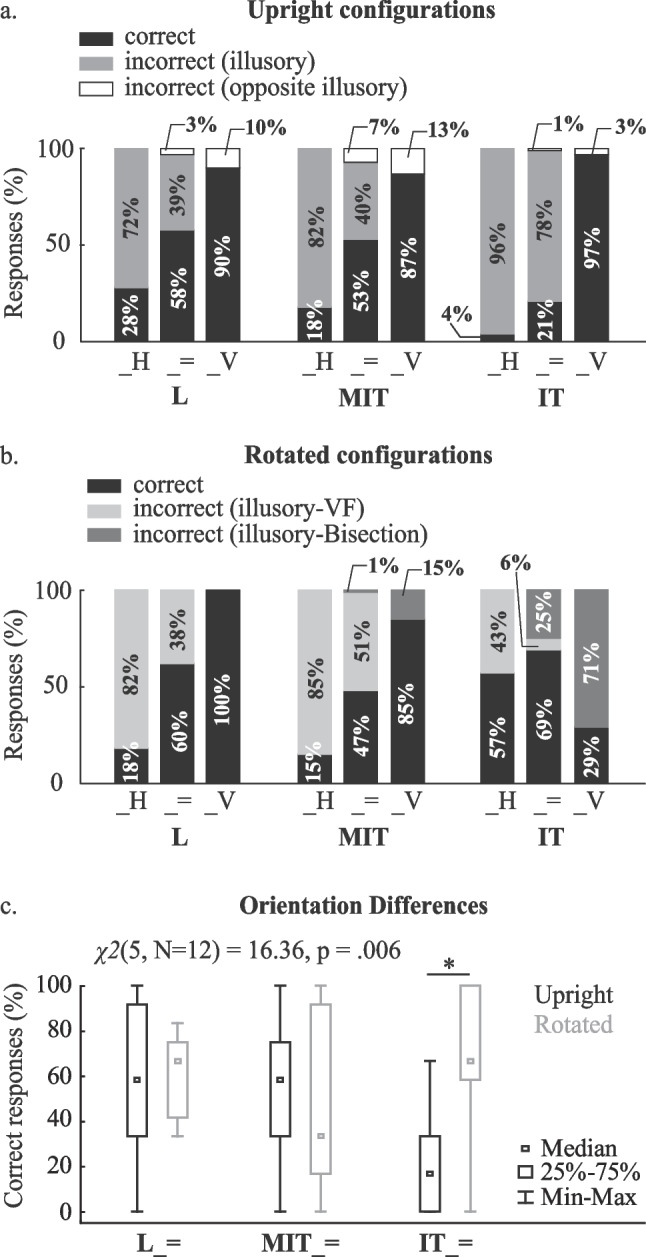
Results of the perceptual judgment task. The average percentages of correct and incorrect responses across all participants for upright (**a**) and rotated (**b**) configurations are shown. Incorrect illusory responses represent incorrect responses by participants that were biased by vertical-horizontal (V-H) illusion with visual field (VF) and bisection effects together (**a**) or independently (**b**) shown. Note that opposite illusory incorrect responses do not exist for upright L, MIT, and IT with a longer horizontal segment (_H), that correct responses for upright _V also coincide with illusory responses, that correct responses for rotated _H also coincide with bisection responses, and that correct responses for rotated _V also coincide with visual field responses. Thus, differences in correct responses were only assessed for configuration orientation (Upright, Rotated) and configuration type (IT, L, MIT) with equal segment lengths (**c**). A Friedman ANOVA test revealed that percentages of correct responses differed across configuration type and orientation. Wilcoxon matched pairs tests revealed only a significant difference between upright and rotated IT. _H represents a configuration involving a longer horizontal than vertical segment; _= represents a configuration involving equal horizontal and vertical segment lengths; _V represents a configuration involving a longer vertical than horizontal segment. *MIT* modified inverted-T, *IT* inverted-T

Percentages of the perceptual judgment task for upright and rotated configurations separated by configuration symmetry are shown in Figs. 4a and b, respectively. These plots show that on average participants produced fewer perceptual inaccuracies for rotated IT than upright IT, while mean perceptual inaccuracies for rotated L and upright L as well as rotated MIT and upright MIT were similar. For upright configurations, illusory perceptions for IT exceeded MIT in 9/12 participants and L in 10/12 participants, while illusory perceptions for L exceeded IT in 10/12 participants for rotated configurations. On average participants produced very similar illusory perceptual judgments between rotated and upright L configurations, as visual field remained the same and no segment bisection existed to expect a difference. Interestingly, percentages of visual field illusory influences between rotated and upright MIT also produced very similar illusory perceptual judgments, while the mean percentage of illusory influences for rotated IT decreased when the horizontal segment length was longer than or equal to the vertical segment length (more correct responses) in all participants and increased when the vertical segment length was longer than the horizontal segment length in 11/12 participants (more incorrect responses). Data analyses confirmed that the percentage of correct responses for rotated IT_= significantly exceeded that for upright IT_= (Fig. 4c). Also of interest, responding “equal” for more than 80% of incorrect perceptual judgments existed for rotated IT.

### Perceptuomotor task

**Fig. 5:**
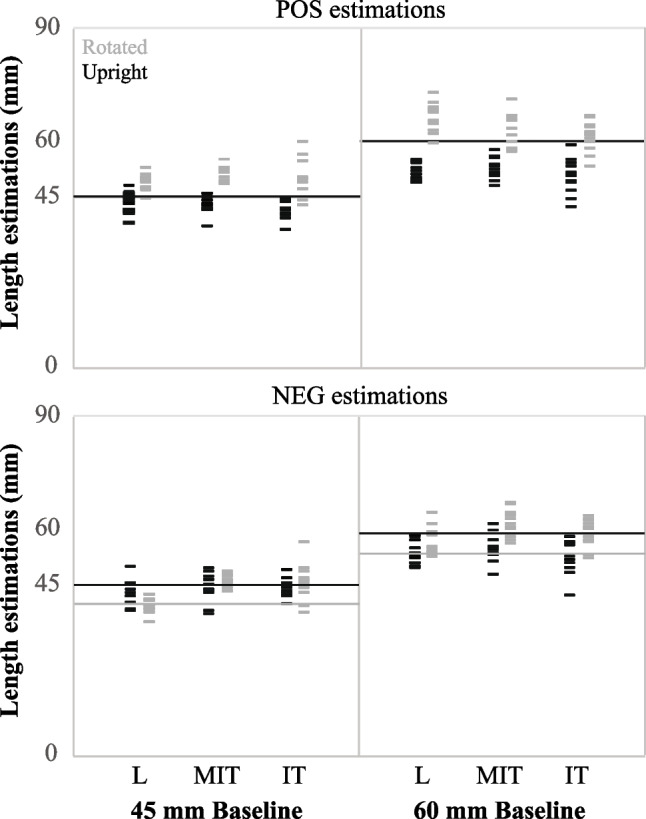
Small dashed segments (tick marks) represent manual length estimations for each experimental trial of one representative participant with individual data similar to mean outcomes. Data for upright (black) and rotated (gray) configurations of the 45-mm (left panels) and 60-mm (right panels) baseline lengths for each trial are shown for positively proportional (POS) estimations (top panels) and negatively proportional (NEG) estimations (bottom panels) with configurations (L, MIT (modified inverted-T), IT (inverted-T)) presented on the x-axis. Long horizontal lines represent the average displacement in control trials for rotated (gray) and upright (black) configurations. Note that the long horizontal lines for rotated configurations (gray) are not visible for POS estimations in the figure because the gray and black lines are overlapped

Control and experimental trial data of manual length estimations for one representative participant with outcomes approximating the group average are shown in Fig. 5. For POS estimations, this participant often overestimated vertical baseline lengths for rotated configurations (compare gray tick marks for trial data relative to gray lines for control data, upper panels) and underestimated horizontal baseline lengths for upright configurations (compare black tick marks for trial data relative to black lines for control data, upper panels). These outcomes were less consistent and/or differed for NEG estimations.

Significant main effects of configuration orientation on mean displacement ratio (*F*(1,11) = 9.331, *p* < 0.05, *ES* = 0.46) indicated that the often overestimated length for rotated configurations (1.04) exceeded the commonly underestimated length for upright configurations (0.96). No other significant main effects existed for mean displacement ratio or its variability (*p* > 0.05).

Several significant interactions were observed for mean displacement ratio. We present the significant two-way interactions in Table 1, which offers insight into differences in configuration symmetry and estimation type when accounting for configuration orientation and differences in configuration symmetry when accounting for estimation type. For example, the mean ratio for L and MIT exceeded that for IT only for rotated configurations, regardless of the estimation type (POS, NEG). Ratios for rotated configurations exceeded those for upright configurations only for POS estimations, regardless of the configuration symmetry (L, IT, MIT). Additionally, ratios for POS exceeded those for NEG only in L, and ratios for L and MIT exceeded those of IT only for POS estimations, regardless of configuration orientation (rotated, upright).

**Table 1:** Results of two-way repeated ANOVA analyses

Comparisons	Main effects (ANOVA)	Post-hoc comparison
Configuration symmetry * Configuration orientation	F(2, 22) = 6.16, p < .01	IT < L, MIT for Rotated
Estimation type * Configuration orientation	F(1, 11) = 5.34, p < .05	Upright < Rotated for POS
Configuration symmetry * Estimation type	F(2, 22) = 8.09, p < .01	POS > NEG for L IT < L, MIT for POS

Configuration symmetry includes L, MIT, and IT configurations; Configuration orientation includes Upright and Rotated configurations; Estimation type includes POS and NEG estimations. Abbreviations: L, MIT—modified inverted-T, IT—inverted-T; POS, Positively proportional estimates; NEG, Negatively proportional estimates

**Fig. 6:**
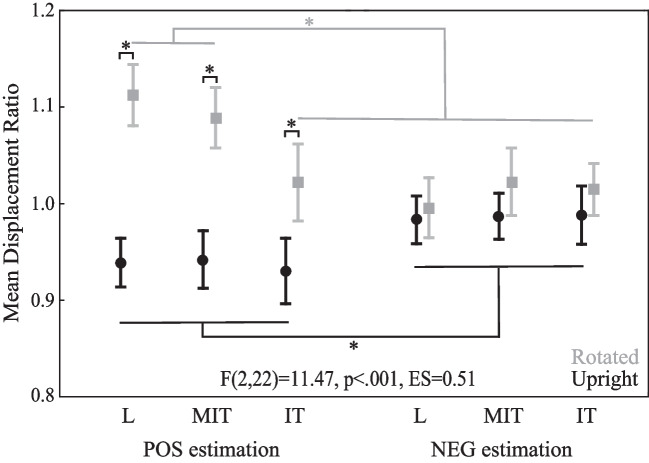
Mean displacement ratios associated with manual length estimations for the configuration orientation (upright-black, rotated-gray) * configuration type (L, MIT (modified inverted-T), IT (inverted-T)) * estimation type (positively proportional (POS) estimation-left panel, negatively proportional (NEG) estimation-right panel) interaction are shown. Significant differences between means are identified by asterisks

However, the significant three-way interaction of configuration symmetry * estimation type * configuration orientation revealed the main results of this study (Fig. 6). Results from Tukey's post hoc test indicated that the ratio for L and MIT exceeded that for IT only for POS estimations of rotated configurations and that ratios of L, MIT, and IT for rotated configurations exceeded upright configurations for POS estimations (p < 0.001 for all; compare gray and black values for POS). Figure 6 also reveals that ratios for each configuration for NEG estimations, whether upright or rotated, and rotated IT configurations for POS estimations were close to 1 and not significantly different from each other, suggesting similar performance to control conditions for these combinations. Except for IT when configurations were rotated (p = 0.99), ratios for POS estimations exceeded those for NEG estimations of rotated configurations (p < 0.001 for L and MIT; compare values in gray for POS and NEG), while ratios for NEG estimations exceeded those for POS estimations of upright configurations (p < 0.01 for L and MIT, p < 0.001 for IT; compare values in black for POS and NEG). These data show that the POS estimations for rotated L and MIT exceeded values greater than 1, while POS estimations for upright L, MIT, and IT were smaller than values less than 1, indicating differences from control trials. These results suggest differences between estimation type for most configuration symmetries and orientations.

## Discussion

The primary aim of this study was to investigate potentially deceptive illusory influences on positively and negatively proportional manual length estimations using curved-path point-to-point movements. We first investigated whether participants recruited for this study succumbed to the V-H illusion perceptually and discussed how these results compare with others in the literature. We then addressed the primary hypothesis that the perceptuomotor control for manual length estimations was not resistant to V-H illusory influences under specific conditions and discussed how the evidence improves our understanding of the associated sensorimotor control.

### Perceptual judgment task

To determine the V-H illusory effects on visual perceptions in our participants, we conducted perceptual judgment experiments involving the three configurations in Figs. 1b, c, and d with the vertical segment length greater than, equal to, or less than the horizontal segment length. For upright configurations, results support the idea that V-H illusory effects on perception followed visual field and bisection influences. As expected, the non-bisected, vertical segment frequently appeared longer than its actual length in this study. Participants also reported illusory responses for IT most often and fewer for L (Fig. 4a), similar to experiments elsewhere, in which participants were forced to choose vertical or horizontal for equal length segment configurations (Künnapas, 1955a; Renier et al., 2006). The reported number of illusory responses for MIT remained close to L for upright configurations, yet opposed other results in which participants reported the fewest illusory responses on average for unsymmetrically bisected upright configurations projected in a vertical plane (Charras & Lupiáñez, 2010; Wolfe et al., 2005). Potential differences in illusory influences on perceptual judgments across studies might result from presentation in the almost horizontal plane over the vertical plane presentation and/or the provision of an “equal” response option, thus three choices, rather than a two-option, forced-choice response (Charras & Lupiáñez, 2010; Mamassian & de Montalembert, 2010; Renier et al., 2006; Wolfe et al., 2005), the latter of which may lead to a response bias (Spence et al., 2001). Although these outcomes for upright configurations do not begin to differentiate support for a given influential construct of visual illusions on perception, they did show V-H illusory influences for this orientation in our participants.

Rotated configurations helped differentiate visual field influences from bisection influences on V-H illusory perceptions. Without symmetrical bisection, configurations influenced perceptual judgments for upright and rotated configurations similarly and primarily followed visual field influences (see similarities between upright and rotated L and upright and rotated MIT, Fig. 4). Furthermore, almost half the incorrect responses when horizontal segment lengths exceeded vertical segment lengths for rotated IT still revealed visual field influences (see IT_H, Fig. 4b). In contrast, percentages of illusory effects decreased and percentages of correct responses increased when presenting participants with rotated IT, and horizontal segment lengths equaled (IT_=) or exceeded (IT_H) vertical segment lengths. For rotated IT, percentages of illusory responses explained by bisection exceeded those for correct responses when vertical segment lengths exceeded horizontal segment lengths (IT_V) to support greater bisection influences for rotated IT for most participants observed in studies with vertical plane projections (Charras & Lupiáñez, 2010; Renier et al., 2006). However, review of actual responses for rotated IT offers greater insight into support for use of different illusory constructs.

The often “equal” length reports for incorrect responses for rotated IT supports the notion that a conflict exists between visual field and bisection illusory influences (Finger & Spelt, 1947). Since ambiguous stimuli, such as those associated with visual illusions, produce a greater theta response in higher brain areas than unambiguous stimuli, people may rely more on ambiguous stimuli for conflict resolution (Mathes et al., 2014). Individual interpretation of illusory conflict and the possibility of additive factors to the conflict likely explain the idiosyncratic differences on perceptual judgments (Charras & Lupiáñez, 2009, 2010; Williams & Enns, 1996; Wolfe et al., 2005). One previous model supported the use of two additive factors, visual field and bisection influences, on perceptions for upright presentations of the V-H illusion in a vertical plane (Mamassian & de Montalembert, 2010). Data on rotated configurations from the present study revealed the existence of greater visual field than bisection influences on V-H illusory perceptions for MIT configurations, regardless of the unsymmetrical bisection that exists. The fact that V-H illusory perceptions can also reverse with configuration rotation here (compare upright and rotated IT results, Fig. 4c) and in a vertical plane (Renier et al., 2006) provides evidence that the central nervous system accounts for different illusory influences associated with configuration symmetry and orientation within the visual field.

### Perceptuomotor task

Results of the present study clearly demonstrated V-H illusory effects on manual length estimations, thus perceptuomotor control, for POS estimations. These results conflict with others which revealed no illusory influences of perpendicular line segment drawings to estimate the main segment length of the Brentano illusion (Fig. 1a; de Grave et al., 2004). Since participants in these studies used positively proportional movements, we considered that conflicting outcomes possibly resulted from the movement dimensionality differences and/or the type of illusion used in each study. Making straight-path point-to-point movements, in which actual movement distance matches movement estimation, may vary from the curved-path point-to-point movements (Izawa et al., 2008), in which movement distance exceeds movement displacement and the associated manual estimation. These dimensionality differences could affect the planning of movement, thus having different outcomes between studies. Results from other studies using the V-H illusion revealed overestimations of vertical baseline lengths for rotated configurations, similar to the present study, to support the possibility that type of illusion may explain the differences between studies. The relative overestimations of vertical to horizontal segment lengths also occurred whether participants blocked portions of long vertical segments of L and IT to match the length of a given horizontal segment (Brosvic et al., 1997; McBride et al., 1987), used a mouse to draw a vertical or horizontal segment of an L or IT to match the length of a given perpendicular segment (Gavilán et al., 2017), or used a keyboard or knob to adjust a horizontal or vertical segment length of L (Künnapas, 1957a, b, c; Richter et al., 2007) or non-connecting segments (Prinzmet al., & Gettleman, 1993) to match its perpendicular length. Knowing that Brentano and V-H illusions can stimulate different brain areas (Axelrod et al., 2017) provides additional support for study outcome differences based on type of illusion.

The observed decrease in displacement for POS estimations associated with mean ratios smaller than 1 could result if participants’ estimations matched a perceived shorter horizontal baseline. Similarly, the increase in displacement for POS estimations associated with mean ratios greater than 1, especially for rotated MIT and L, could result if participants’ estimations matched a perceived longer vertical baseline. The present results support the existence of V-H illusory perceptions corresponding to relatively longer vertical segments than horizontal segments for L (de Montalembert & Mamassian, 2010) and MIT (Wolfe et al., 2005) configurations, regardless of direction (de Montalembert & Mamassian, 2010), and support the use of visual field influences (Künnapas, 1957a, b, c) on perceptuomotor control. Visual field influences produced a relatively strong impact on manual estimations of perpendicular segment lengths regardless of the symmetrical frame used, which has been shown to reduce the perceptual effect of an elliptical visual field (Künnapas, 1959) at least slightly (Gavilán et al., 2017; Houck et al., 1972). The displacement ratio results which approximated 1 for rotated IT could result if the participant’s estimations matched a perceptual conflict between visual field and bisection influences (see more detailed discussion below). Participants revealed greater displacement ratios, thus less expected conflict in illusory influences, for rotated L with no bisection influence and MIT with less bisection influence compared to IT (see Fig. 4c) to support this conflict and indicate greater visual field influences over POS estimations for rotated L and MIT. The configuration symmetry and orientation influences observed in the present study emphasize the importance of human eye location associated with visual field on perceptuomotor control. The fact that these influences existed only for POS estimations suggests the influence of additional mechanisms on such control.

Expected illusory perceptions did not always match perceptuomotor performances. For upright configurations participants underestimated baseline length and displaced the hand less than control trials for POS estimations, yet outcomes did not differ by configuration symmetry. Although the smallest mean displacement ratio was observed for upright IT, this value did not differ significantly from upright MIT or L, like those common for perceptual judgements (Wolfe et al., 2005) and for POS estimations of rotated configurations. These results leave us to wonder why illusory influences on the human perceptuomotor control system differed by the symmetry of upright and rotated configurations for POS estimations. We reasoned that the possibility for differences between manual length estimations of configuration orientations by symmetry involves application of perceptual conflict. Having visual field and bisection influences in the same direction for upright configurations produces no perceptual conflict, thus participants frequently underestimated baseline lengths for IT by 7% on average. These results support others who reported 7% (Finger & Spelt, 1947) and 17% (Gavilán et al., 2017) length underestimations of the horizontal baseline segment. In contrast, having perceptual influences which compete may make the use of each influence more important, thus imperative for control. Results provided evidence for the existence of competing influences on control that imitated those for perceptual judgments at least in part. In this case, bisection influences competed with influences of visual field for symmetrical configurations (Gavilán et al., 2017) and cancelled each other to reveal ratios closer to 1 than MIT and L (see mean ratio for rotated IT, POS estimation, Fig. 6). Although our participants slightly overestimated the vertical baseline lengths by 2% for rotated IT, underestimations also existed (Fig. 5). Participants also underestimated vertical baseline lengths by 3% when drawing horizontal segments (Gavilán et al., 2017) and overestimated vertical baseline lengths by 3% when adjusting the size of a horizontal segment length by pulling on one end to hide part of the segment behind a screen (Finger & Spelt, 1947). These data support the application of multiple illusory constructs to explain manual length estimations of perpendicular segments for this V-H illusory task for upright and rotated configurations.

Unlike POS estimations, mean displacement ratios did not significantly vary among configuration symmetries or orientations for NEG estimations. We considered the varied start locations, thus workspace locations, to explain estimation type differences. Alterations in movement distance in the anterior–posterior directions can vary with start position when reaching toward a given target without accurate feedback of hand position (Sainburg et al., 2003). However, the workspace location hypothesis based on varied start location does not explain why differences existed for upright and rotated configurations that included medial–lateral directions in our study. We also considered that finger position at the start of each manual length estimation for POS estimations began at the configuration intersection, and thus could block a portion of the baseline that participants were estimating. For NEG estimations, in which the finger started at the screen edge, it did not. If blocking baselines for POS estimations encouraged illusory underestimations or overestimations, an estimation shift in a single direction for upright and rotated configurations would exist. Our data revealed underestimations and overestimations and not a single directional shift to support this possibility. Another consideration for differences between performance in NEG and POS estimations may relate to distractors in the workspace. Initiating movement for NEG estimations eliminated view of the finger on the configuration intersection to produce fewer workspace distractions, known to limit illusory influences over performance (de Fockert et al., 2001; Foster & Franz, 2014), possibly by encouraging greater finger-based or egocentric frames of reference (Goodale, 2014; Schenk, 2006). Lastly, we considered control strategies participants could use to accomplish each task: finger displacement or final finger endpoint location. If controlling displacement, extent of movement for POS estimations would approximate baseline lengths, while extent of movement for NEG estimations would require spatial subtraction to approximate baseline lengths. If controlling movement to an endpoint location, participants would estimate an endpoint location based on an approximated baseline length distance from the configuration intersection for each estimation type. Researchers showed evidence for greater stability for length estimations and greater decay of position information for movements with limited online visual feedback to indicate that visuomotor control strategies differ for estimating length and end position (Hesse et al., 2016a, b), with length estimations more susceptible to illusory influences (Hesse et al., 2016a, b; Predebon, 2005). The strategy of estimating endpoints for NEG estimations rather than length would explain the differences observed in the present study, especially when one considers that use of these different strategies would encourage alterations in gaze direction, known to influence manual length estimations (Yan & Hondzinski, 2021), and that endpoint estimations can occur in an egocentric frame of reference (Bernier & Grafton, 2010).

Researchers presented evidence that illusory perceptual biases on action utilize allocentric frames of reference (Schenk, 2006), while attenuation of illusory perceptual biases on action utilize egocentric frames of reference according to its definition (Goodale, 2014). Use of egocentric reference frames, which is considered mandatory for a dissociation between perception and action to occur (Wraga et al., 2000), would encourage fewer illusory influences over actions, like manual length estimations occurring in our NEG estimation condition, and support the two-visual-system hypothesis. Whether participants estimated endpoints with the seen hand, which can occur in an egocentric frame of reference (Bernier & Grafton, 2010; Henriques et al., 1998), and/or utilized a workspace with few distractions, which may also encourage use of an egocentric frame of reference, remains unknown. Whichever strategy participants used in our NEG estimation condition, it differed from the manual length estimations occurring in our POS estimation condition, in which estimating lengths probably occurred in an allocentric frame of reference to encourage greater illusory influences over actions (Goodale, 2014; Schenk, 2006). Therefore, we determined that participants used different control strategies to estimate baseline lengths for positively and negatively proportional movements.

## Limitations

The major limitation of this study is linked to the lack of direct comparison between perceptual task and perceptuomotor task. We chose to accept this limitation to offer directional assistance using the extended segment, which did not allow for direct segment length comparisons during the perceptuomotor task.

## Conclusion

Manual estimations of perpendicular segment lengths corresponded to different aspects of visual perception influences. V-H illusory effects of configurations differed by orientation to show support for varied use of visual field and bisection effects on perceptual judgments. V-H illusory effects of configurations differed by orientation to show support for varied use of visual field and bisection effects on positively proportional manual length estimations. Evidence supported use of visual field and bisection influences on manual length estimations existed when in conflict during movements associated with rotated IT. Positively proportional manual length estimates for configurations with unsymmetrical or no bisection were dominated by visual field influences. Producing positively proportional movements likely encouraged a displacement control strategy and greater use of allocentric programming by the central nervous system, thus an illusory influence existed. Producing negatively proportional movements likely encouraged use of an endpoint control strategy and greater use of egocentric programming by the central nervous system, thus limiting illusory influences on perceptuomotor performance. We conclude that given the correct performance parameters, exploitation of simple 2D illusory influences can guide upper limb sensorimotor control.

## Data Availability

Data of this study will be available upon request from the corresponding author.

## Code availability

Codes used for data analysis are available from the corresponding author, when requested.
